# Research Progress of Pyroelectric Nanogenerator and Its Hybrid Nanogenerators

**DOI:** 10.3390/ma19132823

**Published:** 2026-07-02

**Authors:** Yujia Liu, Shujia Wang, Zongqiang Gao, Hui Zhang, Faqi Zhan, Kun Zhao

**Affiliations:** 1 School of Materials Science and Engineering, Lanzhou University of Technology, Lanzhou 730050, China; 15134043545@163.com (Y.L.);; 2 State Key Laboratory of Advanced Processing and Recycling of Nonferrous Metals, Lanzhou University of Technology, Lanzhou 730050, China

**Keywords:** pyroelectric materials, pyroelectric nanogenerators, hybrid nanogenerators, applications, challenges, prospects

## Abstract

Pyroelectric nanogenerators (PyNGs) have attracted extensive attention for converting thermal energy into electricity, yet their low output power remains a critical bottleneck hindering practical use. This review summarizes various pyroelectric materials and device structures, elucidates the working principle, and discusses their output performances and application scenarios. The correlation between device output and key factors, including intrinsic material properties, electrode dimensions, and external thermal excitation, is systematically examined. Hybrid nanogenerators (HNGs) that couple pyroelectric with piezoelectric, triboelectric, and photovoltaic effects are also reviewed. In addition, the evaluation criteria for pyroelectric energy conversion efficiency are examined, highlighting the need for more systematic studies in this aspect. Finally, key challenges and corresponding strategies are discussed to facilitate the practical deployment of PyNGs in areas such as wearable electronics and self-powered sensors.

## 1. Introduction

With the rapid development of the Internet of Things (IoT) [1,2,3,4,5], the demand for various micro-nanodevices is quite urgent. There are multiple kinds of energy in the environment around us, including heat energy [6,7,8], wind energy [9,10,11], wave energy [12,13,14], droplet energy [15,16,17], vibration energy [18,19,20], and so on. Nanogenerators can not only harvest this energy and convert it into electrical energy, but also offer many advantages such as being lightweight, compact, low-cost, and pollution-free. The energy-harvesting devices mainly include triboelectric nanogenerator (TENG) [21], piezoelectric nanogenerator (PENG) [22], pyroelectric nanogenerator (PyNG) [23], thermoelectric generator (TEG) [24], and solar cell [25]. Among these, the PyNG has attracted particular attention due to its ability to harvest thermal energy from ambient temperature fluctuations. The PyNG operates based on the pyroelectric effect, which converts thermal energy into electrical energy through the rate of temperature change. The pyroelectric current can be expressed as *I* = *P*_c_·*A*(*dT*/*dt*), where *P*_c_ is the pyroelectric coefficient, *A* is the electrode area, and *dT*/*dt* is the temperature change rate [26].

Meanwhile, nanogenerators also have limitations. For example, a single nanogenerator can only harvest a single type of energy from the environment. For this reason, it is hoped that by combining one or several nanogenerators, not only can a variety of energies in the environment be harvested, but the electrical output performance can also be enhanced. By optimizing materials, improving device structures, and designing hybrid nanogenerators (HNGs) to harvest various types of energy from the environment, these advancements can significantly benefit applications in the IoT, self-powered electronic devices, and smart sensing systems.

This article outlines the advancements in pyroelectric materials and device structures over the past several years, covering the working mechanism of PyNGs, the optimization of materials and device designs, and the resultant output performance across various material systems, from inorganic ceramics (such as ZnO, BaTiO_3_ (BTO), KNbO_3_, and PZT) to organic polymers (including polyvinylidene fluoride (PVDF) and poly (vinylidene fluoride-co-trifluoro ethylene) (P(VDF-TrFE))). Finally, typical applications of PyNGs and HNGs are introduced, including liquid crystal displays (LCDs), LEDs, sensors, and self-powered protection systems.

## 2. Pyroelectric Materials and PyNG Device Structures

### 2.1. Pyroelectric Materials

With the development of the pyroelectric effect, more and more pyroelectric nanomaterials have been studied. Here, we select various types of pyroelectric nanomaterials for discussion and summary. The nanostructure and dense interface of pyroelectric materials are critical for improving the output performance of PyNGs.

**Figure 1:**
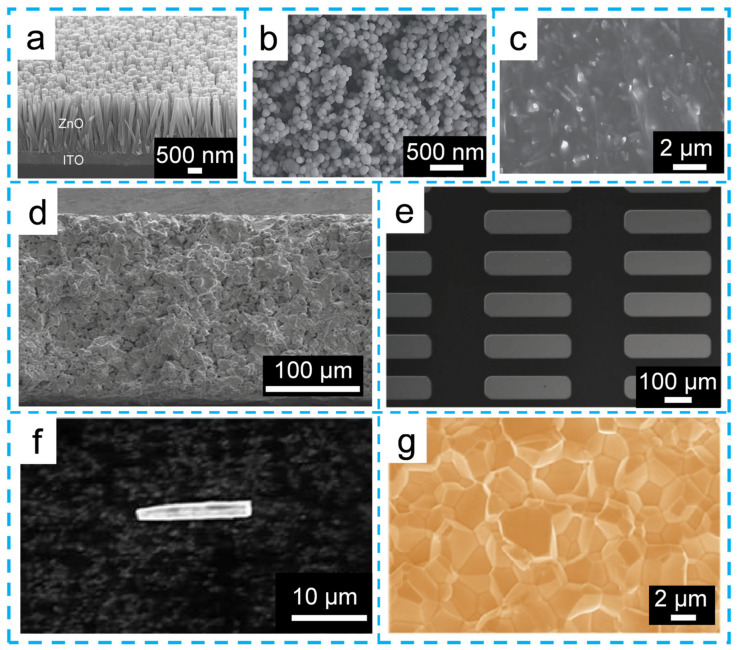
(**a**) Cross-sectional SEM image of a ZnO nanowire array. Reprinted with permission from Ref. [27]. (**b**) SEM image of BTO nanoparticles. Reprinted with permission from Ref. [28]. (**c**) SEM image of the enlarged cross-section of KNbO_3_-PDMS composite film. Reprinted with permission from Ref. [29]. (**d**) SEM image of BFO film. Reprinted with permission from Ref. [30]. (**e**) SEM image of PMN-PT ribbons after reactive ion etching and transferred to PET substrate. Reprinted with permission from Ref. [31]. (**f**) Surface SEM image of a single PZT nanowire. Reprinted with permission from Ref. [32]. (**g**) Cross-sectional SEM image of a PZT film. Reprinted with permission from Ref. [33].

ZnO is one of the most representative pyroelectric materials, with the dual properties of piezoelectricity and semiconductivity. Its dual properties offer significant potential for applications in piezotronics and piezophototronics. Excellent pyroelectric properties of ZnO nanomaterials, such as nanobelts, nanorod arrays, and nanoparticles (NPs), have been confirmed in numerous reports. ZnO nanowire arrays were synthesized on an indium tin oxide (ITO) substrate using solution growth technology. Figure 1a displays a cross-sectional scanning electron microscope (SEM) image of ZnO nanowires, which have an approximate length of 2 µm [27]. BTO not only has good ferroelectricity but is also an outstanding pyroelectric nanomaterial. Due to its excellent properties, BTO has been extensively studied and reported. Figure 1b shows the SEM image of BTO nanoparticles [28]. It can be seen that the BTO nanoparticles exhibit good uniformity and dispersibility, with a diameter of approximately 100 nm. Similar to BTO, (Bi_0.5_Na_0.5_)TiO_3_ (BNT) is another pyroelectric material. Additionally, Figure 1c presents a cross-sectional SEM image of the KNbO_3_ nanowire–polydimethylsiloxane (PDMS) composite film, revealing that the nanowires are randomly oriented and uniformly dispersed within the polymer matrix without obvious aggregation [29]. Figure 1d displays the cross-sectional SEM image of the sintered BiFeO_3_ (BFO) film [30], illustrating its good compactness and a thickness of approximately 220 μm. Figure 1e presents the SEM image of the 0.7Pb(Mg_1/3_Nb_2/3_)O_3_-0.3PbTiO_3_ (PMN-PT) tape structure after reactive ion etching and transfer to a polyethylene terephthalate (PET) substrate, with Au/Ti as the bottom electrode [31]. Figure 1f shows an SEM image of a single PZT nanowire, where the diameter and length are 2 μm and 10 μm, respectively [32]. Similarly, Figure 1g exhibits an enlarged SEM image of the PZT film [33]. The image reveals that the PZT film is composed of numerous crystal grains and has a dense structure.

Among the materials shown in Figure 1, PZT and PMN-PT are lead-based ceramics. These materials are inherently toxic and can cause significant harm to both the environment and human health during fabrication, service, and disposal. Their use is increasingly restricted by international environmental regulations, most notably the EU RoHS Directive. As a result, developing high-performance lead-free piezoelectric ceramics as viable substitutes has become a major focus in this field. Promising candidate materials include BNT, KNbO_3_ and CuInP_2_S_6_ (CIPS) [34], all of which have demonstrated competitive pyroelectric performance in recent reports. BNT exhibits competitive pyroelectric coefficients and a relatively high depolarization temperature. KNbO_3_ offers a high Curie temperature alongside environmental friendliness. CIPS is a room-temperature ferroelectric with promising pyroelectric properties and a lead-free composition. Nevertheless, lead-free ceramics still suffer from several drawbacks relative to their lead-based counterparts, including lower pyroelectric coefficients, higher dielectric losses, and thermal instability near the depolarization temperature. Addressing these issues through compositional tuning and phase boundary engineering remains a critical research direction.

**Figure 2:**
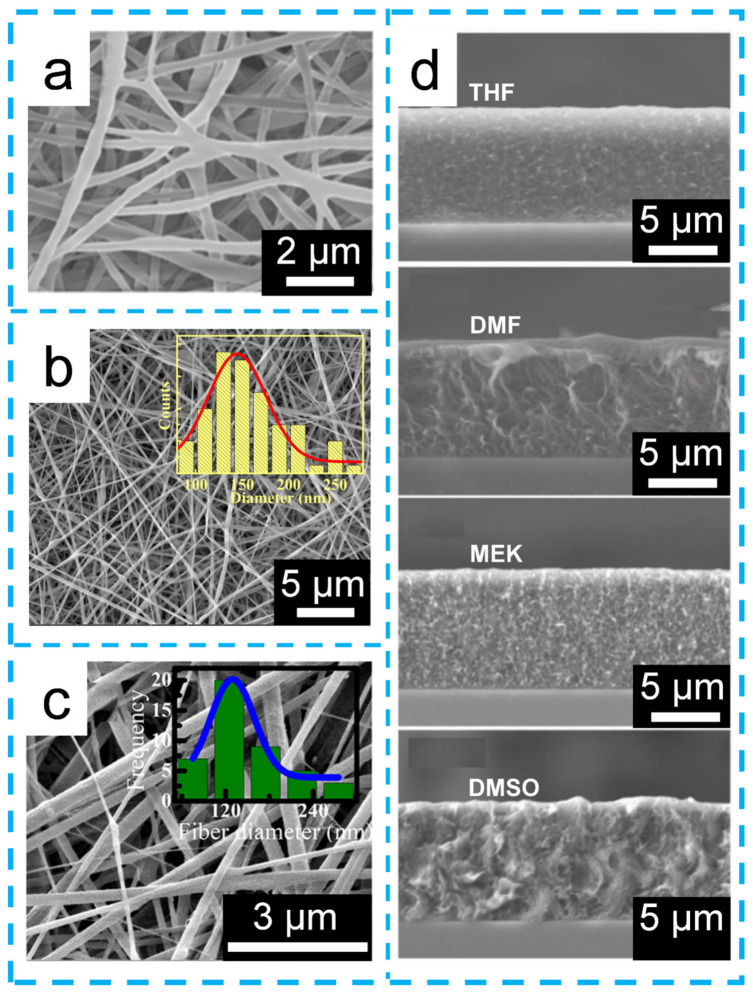
(**a**) SEM image of PVDF nanowires. Reprinted with permission from Ref. [37]. (**b**) FE-SEM image of PVDF-MAPI nanofibers and their diameter profiles. Reprinted with permission from Ref. [40]. (**c**) FE-SEM image of PVDF/1 wt% GO fibers and their diameter profiles. Reprinted with permission from Ref. [41]. (**d**) Cross-sectional FE-SEM images of P(VDF-TrFE) films fabricated with the solvents THF, DMF, MEK, and DMSO. Reprinted with permission from Ref. [42].

PVDF and P(VDF-TrFE) are also excellent pyroelectric materials [35,36]. Figure 2a shows the SEM image of PVDF nanowires [37]. The diameter of the PVDF membranes prepared by electrospinning ranges from 200 to 500 nm. Doping with functional materials is an effective method to further enhance their pyroelectric performance and has been extensively studied [38,39]. Figure 2b exhibits the FE-SEM image of PVDF-methylammonium lead iodide (MAPI) nanofibers along with their diameter distribution map [40]. It can be observed that the nanofibers have a smooth, bead-free surface and an average fiber diameter of 145 ± 72 nm. Figure 2c presents the SEM image of PVDF containing 1 wt% graphene oxide (GO), with an inset displaying the corresponding fiber diameter distribution [41]. These nanofibers are randomly oriented without any signs of agglomeration, with an average diameter of approximately 125 nm. Solvent dipole moments also influence the performance of pyroelectric materials. Figure 2d displays cross-sectional FE-SEM images of P(VDF-TrFE) films dissolved in tetrahydrofuran (THF), dimethylformamide (DMF), methyl ethyl ketone (MEK), and dimethyl sulfoxide (DMSO) [42]. While the film thickness remains approximately 8.5 μm for all four solvents, their cross-sectional morphologies show significant differences.

In order to compare the pyroelectric performance of these diverse materials, key physical parameters, such as the pyroelectric coefficient, Curie temperature, and dielectric constant are summarized in Table 1.

In summary, the main pyroelectric nanomaterials include ZnO, BTO, BNT, KNbO_3_, BFO, PMN-PT, PZT, PVDF, and P(VDF-TrFE). These nanomaterials can be broadly divided into ceramic-based and polymer-based materials. As evidenced by the data in Table 1, these two categories exhibit fundamentally different performance profiles, necessitating distinct optimization strategies. For ceramic materials, the output performance is primarily enhanced by improving purity, optimizing particle size, and increasing material compactness. For polymer-based materials, performance improvement is mainly achieved through different preparation methods, raw material optimization, and nanoparticle doping. From ZnO to BFO, BTO, PZT, and PMN-PT, the pyroelectric coefficients show a clear upward trend (from ~10 to ~1000 µC/m^2^·K), confirming that ceramic ferroelectrics exhibit significantly higher values than polymeric materials. Meanwhile, the high Curie temperatures of BFO and KNbO_3_ suggest their potential in harsh environments, whereas the flexibility of PVDF-based materials makes them ideal for wearable and portable electronics. This quantitative benchmark provides a meaningful reference for selecting appropriate pyroelectric materials for specific nanogenerator and sensor applications.

### 2.2. The Structures of PyNG and HNG

**Figure 3:**
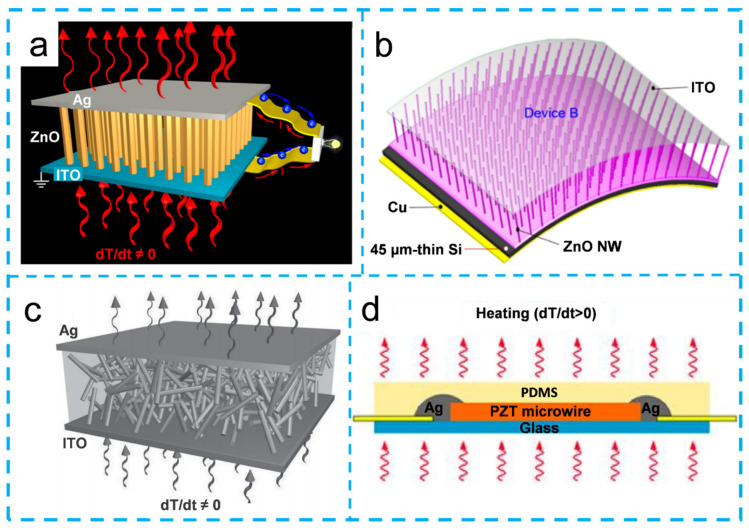
(**a**) Schematic diagram of ZnO nanowire-based PyNG. Reprinted with permission from Ref. [27]. (**b**) Schematic diagram of p-Si/n-ZnO nanowire-based PyNG. Reprinted with permission from Ref. [54]. (**c**) Schematic diagram of KNbO_3_ nanowire-based PyNG. Reprinted with permission from Ref. [29]. (**d**) Schematic diagram of a single PZT nanowire-based PyNG. Reprinted with permission from Ref. [32].

Figure 3 illustrates the representative structures of PyNGs. Figure 3a presents a schematic diagram of a PyNG, which consists of an upper silver (Ag) electrode, ZnO nanowires, a lower ITO electrode, and external circuits [27]. The ZnO nanowire array is formed on an ITO substrate using a solution growth method. Figure 3b displays a schematic diagram of a PyNG that utilizes a 45 μm ultra-thin Si/ZnO nanowire configuration and is activated by near-infrared light [54]. Both devices in Figure 3a,b use the hydrothermal method to synthesize ZnO nanowires, employ ZnO as the pyroelectric material, and use ITO and Ag or Cu as electrodes. The primary distinction is that the device in Figure 3a relies on temperature changes to trigger the pyroelectric effect, whereas the device in Figure 3b uses a p-Si/n-ZnO heterojunction to achieve near-infrared light-triggered pyroelectric performance. Figure 3c shows a schematic diagram of a KNbO_3_/PDMS-based PyNG, in which the volume ratio of KNbO_3_ nanowires to PDMS polymer is 3:7 [29]. The device consists of an upper Ag electrode, a lower ITO electrode, and a middle layer composed of KNbO_3_ nanowires and PDMS polymer. Figure 3d presents a schematic diagram of a PZT-based PyNG [32]. The device structure consists of a thin glass substrate, PZT microwires, Ag paste used to fix both ends of the microwires, and a PDMS encapsulation layer.

**Figure 4:**
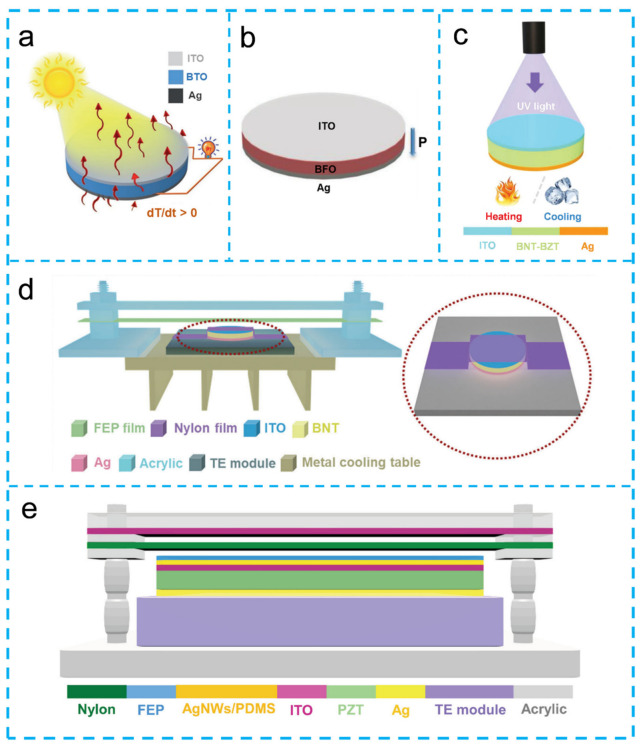
(**a**) Schematic diagram of a BTO-based HNG. Reprinted with permission from Ref. [28]. (**b**) Schematic diagram of a BFO-based HNG. Reprinted with permission from Ref. [30]. (**c**) Schematic diagram of a BNT-BZT-based HNG. Reprinted with permission from Ref. [56]. (**d**) Schematic diagram of a BNT-based HNG. Reprinted with permission from Ref. [47]. (**e**) Schematic diagram of a PZT-based HNG. Reprinted with permission from Ref. [57].

In order to further enhance the output performance, researchers have designed HNGs. The device in Figure 4a uses a BTO film as the pyroelectric and photoelectric active material, with ITO as the top electrode and Ag as the bottom electrode [28]. Zhao et al. enhanced the photocurrent through the ferro-pyro-phototronic effect in BTO for a self-powered flexible photodetector system using a device similar to that in Figure 4a [55]. Figure 4b shows the schematic structure of the ITO/BFO/Ag device [30], where BFO serves as a representative pyroelectric–photoelectric material. Figure 4c displays the schematic structure of the HNG based on 0.94(Bi_0.5_Na_0.5_)TiO_3_-0.06Ba(Zr_0.25_Ti_0.75_)O_3_ (BNT-BZT) ferroelectric material, as reported by Zhao et al. [56]. In this device, BNT-BZT is selected as the pyroelectric component, ITO and Ag are used as electrodes, and the relationship between pyroelectric output and UV light intensity is utilized to design a temperature sensor with UV light regulation. Figure 4d presents a schematic diagram of a device that integrates a photovoltaic cell (PVC), a PyNG, and a TENG into a single unit [47]. The bottom thermoelectric (TE) module provides heating and cooling for the PyNG component. The top BNT ceramic is coated with ITO electrode at the top and Ag electrode at the bottom, and the ITO electrode at the top can absorb light energy for the PVC device. Nylon is attached to the ITO electrode, while the fluorinated ethylene propylene (FEP) film is held in place by an acrylic sheet. The contact–separation motion between the nylon and FEP during movement not only generates triboelectric output but also provides external strain to the BNT ceramic, forming the PiENG module. Figure 4e shows a schematic diagram of a PZT-based single-structure multi-effect coupling nanogenerator [57]. The device consists of three parts: the top part, composed of nylon and FEP, as the TENG; the middle part, consisting of PZT material, serves as the piezoelectric, pyroelectric, and photoelectric component, with a bottom Ag electrode and an upper electrode made of Ag NWs/PDMS combined with ITO; and the bottom part, the TE module supported by the radiator, which mainly provides the temperature source for the middle part.

**Figure 5:**
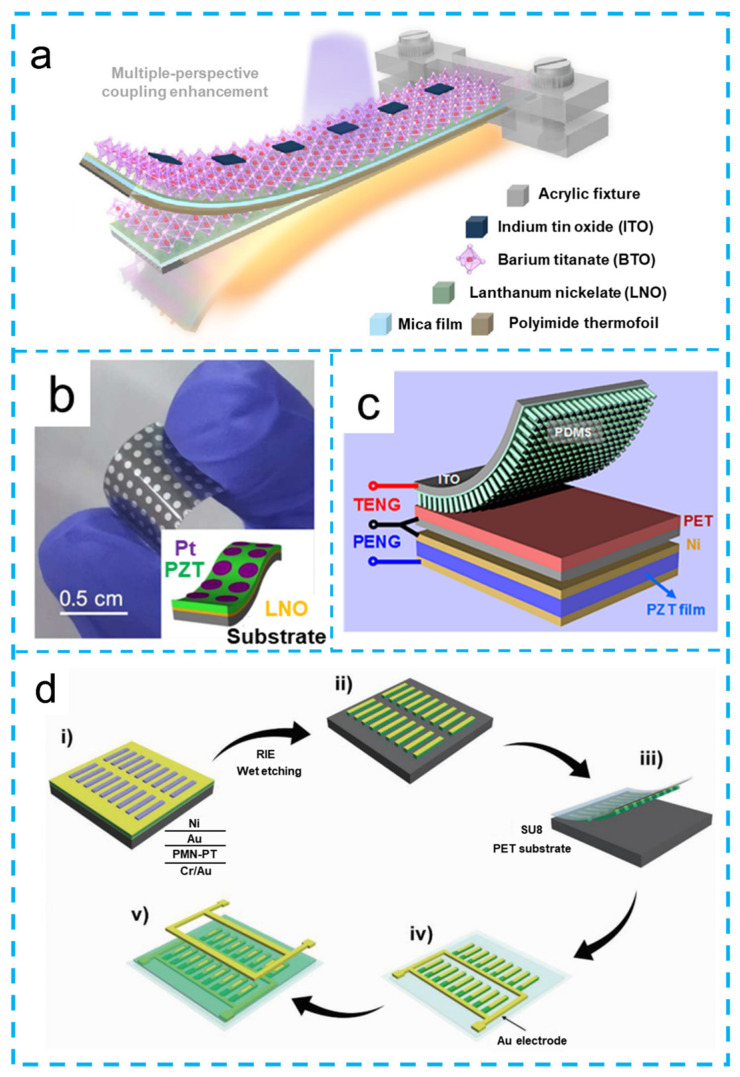
(**a**) Schematic diagram of multi-perspective coupling enhancement in a BTO film-based HNG. Reprinted with permission from Ref. [58]. (**b**) Photo of the PZT film-based HNG. Reprinted with permission from Ref. [59]. (**c**) Schematic diagram of the HNG composed of a PDMS nanowire-based TENG and a PZT thin-film PENG. Reprinted with permission from Ref. [60]. (**d**) Schematic diagram of the preparation process of a PMN-PT tape device structure on a plastic substrate. Reprinted with permission from Ref. [31].

To further expand the application scenarios toward flexible and wearable devices, flexible HNGs have been designed and investigated. Figure 5a presents a flexible, temperature-modulated photovoltaic-coupled nanogenerator based on a ferroelectric BTO thin film [58]. The nanogenerator consists of lanthanum nickel oxide (LNO) and BTO positioned on a mica film, with an ITO electrode deposited on the top. A polyimide thermal foil is attached to the bottom to help control the temperature. Figure 5b presents a photograph of a PZT-based HNG, with the inset showing a schematic diagram of the device [59]. The device is composed of a Ni-Cr metal foil substrate, an LNO bottom electrode, a PZT film, and a Pt top electrode. Notably, LNO exhibits high conductivity and provides uniform nucleation sites for the PZT film. Additionally, the Ni-Cr substrate is flexible and compatible with PZT-based perovskite films. Figure 5c illustrates a hybrid device consisting of a TENG and a PENG [60]. The top TENG part is composed of a transparent ITO film electrode, a flexible PDMS nanowire array layer, and a PET film. The lower PENG section features two Ni electrodes and a PZT film, which also serves as the pyroelectric material. Figure 5d depicts a schematic diagram of the manufacturing process for PMN-PT ribbon devices [31]. The device structure consists of a bottom Cr/Au electrode, a middle PMN-PT layer, and top Au and Ni electrodes, where Ni is used as an etching mask. Through sequential thin-film processing and microfabrication, including transfer printing and selective etching, a compact PMN-PT ribbon array was constructed with a footprint of 3 mm × 3 mm. The final device is configured with a full set of bottom interconnects, isolated top contacts, and a central SU8 dielectric layer to prevent electrical shorting.

In recent years, PVDF-based PyNGs have been widely studied. Most of these devices employ the electrospinning method to fabricate PVDF nanofibers, which serve as both pyroelectric and piezoelectric materials. To further improve the output performance of PyNGs and HNGs, researchers have focused on three main strategies: (i) developing multilayer device structures, (ii) doping PVDF with functional nanoparticles, and (iii) optimizing electrode materials. While the intrinsic properties of PVDF are often preserved, the use of enhanced electrodes and rationally designed architectures has been demonstrated to significantly boost the overall output of PyNGs and HNGs.

**Figure 6:**
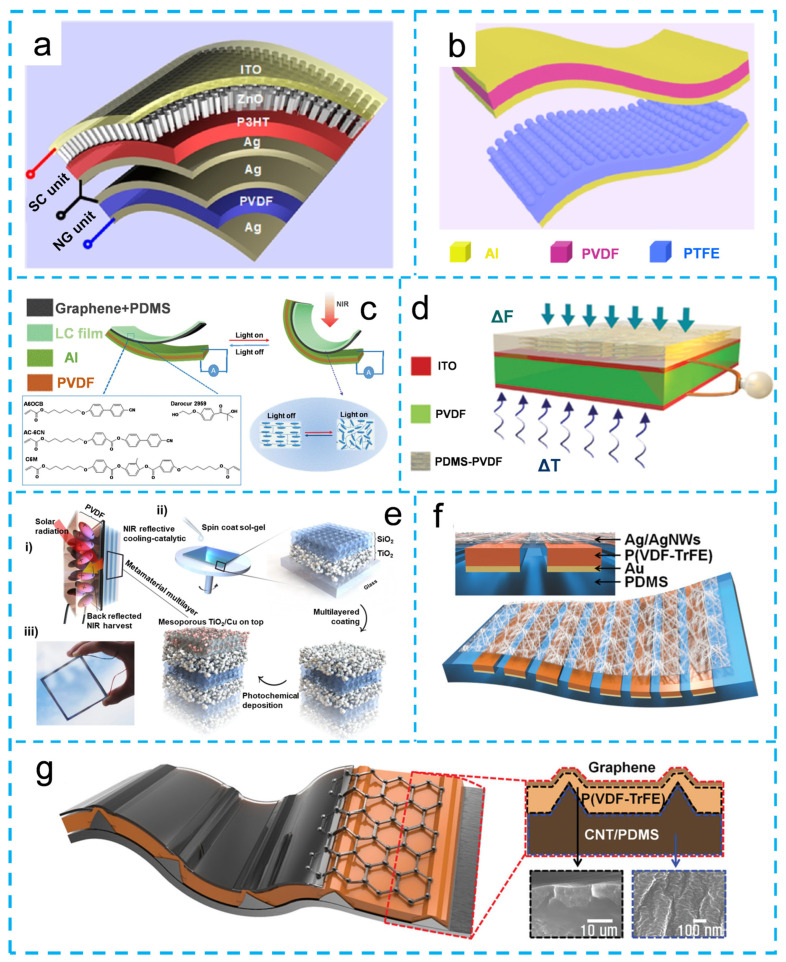
(**a**) Schematic diagram of hybrid device structure of PVDF-based pyroelectric, piezoelectric nanogenerator, and ZnO-P3HT heterojunction solar cell. Reprinted with permission from Ref. [25]. (**b**–**d**) Schematic diagrams of PVDF-based HNGs. Reprinted with permission from Refs. [61], [62] and [37] respectively. (**e**) Schematic diagram of preparation process of PVDF-based thermal nanophotonic-pyroelectric device structure. Reprinted with permission from Ref. [63]. (**f**,**g**) Schematic diagrams of P(VDF-TrFE)-based HNGs. Reprinted with permission from Refs. [64] and [65] respectively.

Figure 6a presents a schematic diagram of a PVDF-based hybrid device [25]. The device consists of a top solar cell and a bottom pyroelectric/piezoelectric nanogenerator. The top solar cell is composed of a transparent ITO electrode, ZnO nanowire arrays, a P3HT film, and an Ag electrode. The bottom HNG comprises two Ag electrodes and a flexible PVDF film. Figure 6b shows a schematic diagram of a flexible PVDF-based HNG [61]. The top part is a pyroelectric/piezoelectric nanogenerator made of a PVDF film and two Al electrodes, while the bottom part is a TENG composed of a PTFE film and Al electrodes. Figure 6c presents the structure of a PVDF-based HNG [62]. The device is composed of liquid crystal elastomer (LCE), graphene-doped PDMS, upper and lower Al electrodes, and a PVDF film. The left inset shows the chemical structure of the LCE, and the right inset displays photographs of the LCE before and after near-infrared (NIR) light irradiation. Figure 6d illustrates a design where PVDF serves as the pyroelectric and piezoelectric material, with a PVDF nanowire–PDMS composite film on top acting as the triboelectric layer, and conventional ITO used as both the upper and lower electrodes of the PVDF film [37]. Figure 6e shows a schematic diagram of the preparation process of a thermal nanophotonic structure [63]. As illustrated in Figure 6e(i), the solar collector PVDF and thermal nanophotonic materials enable simultaneous near-infrared heat reflection, catalysis, and energy harvesting. The thermal nanophotonic structure consists of alternating layers of titanium dioxide (TiO_2_) and mesoporous silicon dioxide (SiO_2_), with a top layer of mesoporous TiO_2_ and copper. These layers are formed by alternating spin coating or dip coating. The ferroelectric PVDF film acts as a solar heat collector, converting temperature changes into electrical output. An optical photograph of the thermal nanophotonic pyroelectric structure is also presented, demonstrating high optical transparency and scratch resistance, which makes it suitable for smart window and display applications. In contrast to the planar structures discussed above, Figure 6f,g feature micro-patterned P(VDF-TrFE) as the pyroelectric and piezoelectric active material. In Figure 6f, the device consists of a thin Ag film and Ag nanowires as the top electrode, Au as the bottom electrode, and PDMS as the substrate template [64]. In Figure 6g, the three-layer structure employs graphene as the top electrode, P(VDF-TrFE) as the active layer, and PDMS-CNT as the bottom electrode [65].

The key improvement in devices lies in the doping of PVDF with functional nanoparticles and the selection of electrode materials. In subsequent studies, a conductive polymer, poly(3,4-ethylenedioxythiophene):poly(styrene sulfonate) (PEDOT:PSS), was used as both the top and bottom electrodes, with CdS-rGO/PVDF nanofibers serving as the pyroelectric active material [66]. Sun et al. developed a PVDF-based device where PVDF functions as both a pyroelectric and a piezoelectric material [67]. PDMS is used as both a packaging material and a friction layer, forming a triboelectric–piezoelectric HNG with PVDF. A notable improvement in this design is the use of transparent electrodes resembling leaf veins as the upper and lower electrodes for PVDF. Gokana et al. developed a pyroelectric device featuring a serpentine electrode (SRE) and modified it using cesium tungsten bronze (Cs_0.33_WO_3_) [68]. In this device, the top electrode is a conductive ink composed of silver nanowires, graphene and Cs_0.33_WO_3_ applied by screen printing. The middle layer is a fiber film made of PVDF and Cs_0.33_WO_3_ fabricated by electrospinning, and the bottom electrode consists of a Ni-Cu electrode.

In summary, PyNGs and HNGs are broadly distinguished by both their material class and their functional complexity. Ceramic-based devices, typically employing inorganic ferroelectrics, deliver superior pyroelectric coefficients, high thermal stability, and robust electrical output, making them suitable for high-temperature scenarios. However, their intrinsic rigid form factor severely restricts applications in wearable or conformable electronics. Polymer-based counterparts, predominantly PVDF and its copolymers, offer excellent mechanical flexibility, lightweight construction, solution processability, and compatibility with large-area fabrication. These attributes make them promising candidates for flexible and wearable devices.

Regarding device architecture, standalone PyNGs harvest energy solely from temperature fluctuations via the pyroelectric effect, providing a straightforward design with minimal interfacial losses but inherently limited energy output. In contrast, HNGs integrate multiple energy-harvesting mechanisms, including pyroelectric, piezoelectric, triboelectric, and even photovoltaic effects, within a single structure. This integration significantly enhances overall power density and enables multifunctional sensing capabilities. Nevertheless, it inevitably introduces greater interfacial complexity, material compatibility issues, and fabrication challenges. Ultimately, the selection among these designs reflects a fundamental trade-off among output performance, mechanical flexibility, and structural simplicity, guided by specific application requirements.

## 3. Working Principle, Output Performance, and Applications of the PyNGs and HNGs

### 3.1. The Working Principle of the PyNG

**Figure 7:**
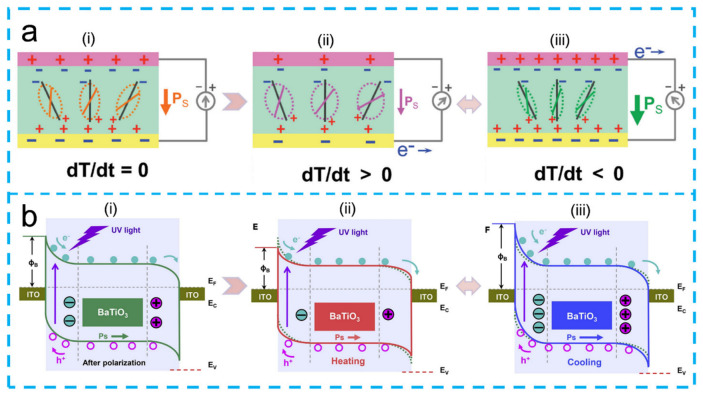
(**a**) The working principle of PyNG, (i) room temperature (*dT*/*dt* = 0); (ii) heating (*dT*/*dt* > 0); (iii) cooling (*dT*/*dt* < 0) conditions. Reprinted with permission from Ref. [57]. (**b**) Diagram of the mechanism of the pyroelectric effect under UV illumination in ferroelectric materials at (i) room temperature (*dT*/*dt* = 0); (ii) heating (*dT*/*dt* > 0); and (iii) cooling (*dT*/*dt* < 0) conditions. Reprinted with permission from Ref. [71].

The process of current generation in a PyNG is illustrated in Figure 7a(i–iii) [57]. As shown in Figure 7a, the device consists of three layers: an upper ITO electrode, a middle layer of PZT as the pyroelectric material, and a lower Ag electrode. As shown in Figure 7a(i), at the initial state, the rate of temperature change is zero (*dT*/*dt* = 0). In this condition, the electric dipoles in the poled PZT material are aligned along the polarization direction, reaching an equilibrium state at a constant temperature. Since the spontaneous polarization remains constant, no electrons are transferred across the electrodes, and no current is generated in the external circuit. As shown in Figure 7a(ii), when heating is applied (*dT*/*dt* > 0), the electric dipoles deflect at a larger angle along their respective alignment axes, leading to a decrease in spontaneous polarization intensity. Consequently, electrons flow from the Ag electrode to the ITO electrode through the external circuit, resulting in a current that flows from the ITO side to the Ag side. Conversely, when cooling is applied (*dT*/*dt* < 0), the electric dipoles oscillate at a smaller angle along their alignment axes, resulting in an increase in spontaneous polarization intensity, as shown in Figure 7a(iii). As a result, the direction of electron flow is opposite to that observed during heating, generating a negative potential in the external circuit.

Building on the above discussion, for a comprehensive understanding of PyNG output, it is necessary to distinguish the effective pyroelectric coefficient into three distinct physical mechanisms. The primary pyroelectric effect arises from the intrinsic temperature dependence of spontaneous polarization under constant strain. It represents the true pyroelectric response of the material and is typically the dominant contribution in dense, rigid ceramics [69]. The secondary pyroelectric effect originates from thermal expansion of the material, which generates mechanical strain. Through the piezoelectric effect, this strain induces an additional change in polarization. This contribution depends on material properties such as elastic stiffness, piezoelectric coefficients, and thermal expansion coefficients [69]. In polymer-based materials such as PVDF, which have larger thermal expansion coefficients and lower elastic moduli, the secondary effect may play a non-negligible role. The tertiary pyroelectric effect arises from non-uniform heating that creates a temperature gradient across the sample. The resulting thermal stress, through the piezoelectric effect, generates an additional polarization response [70]. This effect is particularly relevant in thick samples or under rapid, localized heating conditions, but is often negligible in well-designed devices with uniform thermal excitation.

Figure 7b shows the mechanism of the pyroelectric effect under UV light irradiation in ferroelectric materials [71]. Here, BTO is used as the pyroelectric part and ITO as the electrode. As shown in Figure 7b(i–iii), the polarization charges at the two BTO/ITO interfaces can either increase or decrease the Schottky barrier height, depending on the temperature condition.

At room temperature (Figure 7b(i)), after the polarization of BTO, the opposite polarization charges at the two BTO/ITO interfaces respectively increase one Schottky barrier and decrease the other. Under UV light irradiation, the photoinduced electron–hole pairs can flow effectively between Schottky barriers of different heights. As shown in Figure 7b(ii), when the device is heated, the polarization of BTO weakens, resulting in a decrease in the Schottky barrier height at the left end and an increase at the right end. The change in the Schottky barrier heights weakens the effective separation and directional flow of the photoinduced electron–hole pairs. As shown in Figure 7b(iii), when the device is cooled, the polarization of BTO strengthens, leading to an increase in the Schottky barrier height at the left end and a decrease at the right end. This opposite effect, compared to heating, results in more efficient separation and directional flow of the photoinduced electron–hole pairs. Thus, heating coupled with light irradiation weakens the photocurrent intensity, while cooling coupled with light irradiation enhances it.

This Schottky barrier modulation mechanism has been experimentally validated in ferroelectric materials. Zhao et al. demonstrated that in radially polarized BTO, the ferro-pyro-phototronic effect induces energy band bending that modulates the Schottky barrier height, resulting in a dramatic photocurrent enhancement. Specifically, temperature variation induces pyroelectric polarization, which in turn causes band bending that modulates the Schottky barrier height [71].

### 3.2. The Output Performance of the PyNGs

The pyroelectric current equation *I* = *P*_c_·*A*(*dT*/*dt*) provides a direct analytical basis for understanding the performance disparities among different PyNGs. According to this equation, the output current is governed by three independent parameters: the material’s pyroelectric coefficient, electrode area, and the temperature change rate. This framework allows for a quantitative deconvolution of material-intrinsic and device-design contributions, enabling a more rigorous comparison across diverse material systems and device architectures.

**Figure 8:**
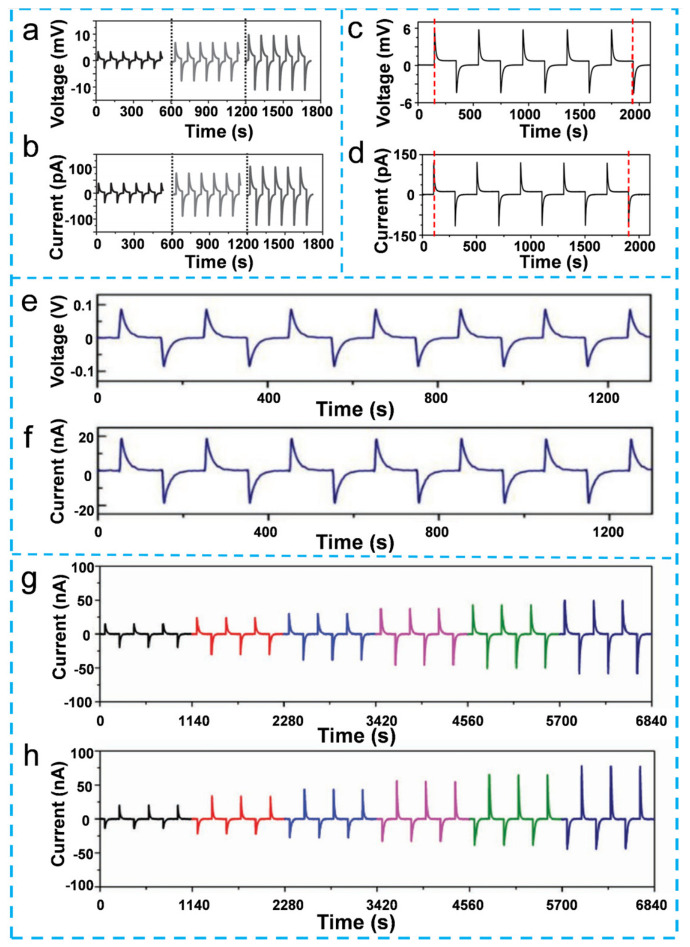
(**a**,**b**) The output voltage and current performance of KNbO_3_ nanowire-based PyNG. Reprinted with permission from Ref. [29]. (**c**,**d**) The output voltage and current performance of ZnO nanowire-based PyNG. Reprinted with permission from Ref. [27]. (**e**,**f**) The output voltage and current performance of PMN-PT ribbon-based PyNG. Reprinted with permission from Ref. [31]. (**g**,**h**) The output currents of BTO-based PyNG under heating and cooling conditions. Reprinted with permission from Ref. [44].

Figure 8a,b show the output performance of a KNbO_3_ nanowire-based PyNG under different temperature changes [29]. Yang et al. reported that under the largest temperature change (about 40 K), the output voltage of the KNbO_3_ nanowire-based PyNG is about 10 mV and the current is about 120 pA. The modest output of the KNbO_3_-PDMS PyNG under large Δ*T* stems from the inherently low pyroelectric coefficient of KNbO_3_ (*P*_c_ ≈ 50 µC/m^2^·K) and the non-contributing PDMS matrix, which reduces the effective pyroelectric coefficient per unit volume of the composite. Figure 8c,d present the output performance of a ZnO nanowire-based PyNG under heating conditions [27]. Yang et al. reported that the output voltage and current of the ZnO nanowire-based PyNG were about 5.8 mV and 120.4 pA, respectively, under a temperature change from room temperature (295 K) to 304 K. The low output is primarily attributed to the modest pyroelectric coefficient of ZnO (*P*_c_ ≈ 12–15 µC/m^2^·K) and the relatively small electrode area typical of nanowire-array devices. Figure 8e,f show the output signals of PMN-PT ribbon-based PyNG [31]. Under a temperature change from room temperature (295 K) to 303 K, the output voltage and current under forward conditions are measured as 0.1 V and 20 nA, respectively. Although it exhibits a high pyroelectric coefficient (*P*_c_ ≈ 1040 µC/m^2^·K), the absolute output of the device is often constrained by the low thermal conductivity of the substrate and packaging materials. Figure 8g,h illustrate the performance of a BTO film-based PyNG [44]. When the temperature change rate varies from −0.85 K/s to 0.98 K/s, the peak current increases from −44.4 nA to 49.8 nA. With *P*_c_ and *A* held constant, this proportional relationship between *I* and *dT*/*dt* is consistent with the pyroelectric current equation.

**Figure 9:**
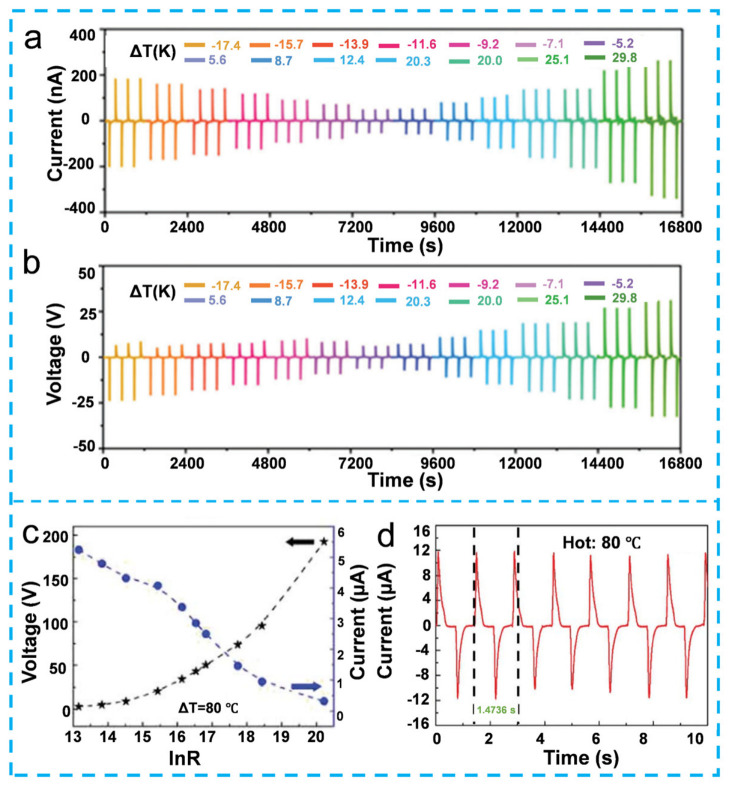
(**a**,**b**) Plot of the output current and voltage of BNT-based PyNG under different temperature variations. Reprinted with permission from Ref. [47]. (**c**,**d**) The output voltage and current of PVDF-based PyNG. Reprinted with permission from Ref. [72].

Figure 9a,b present the output *I*_sc_ and *V*_oc_ of the ITO/BNT/Ag nanogenerator under varying temperature conditions. When the temperature variation (Δ*T*) is −17.4 K, the peak current is −0.201 μA, and the peak voltage is −23.6 V. Conversely, with a temperature variation of 29.8 K, the peak current rises to 0.261 μA, while the peak voltage reaches 31.5 V [47]. Under similar electrode areas and temperature change rates, the higher output of the BNT device is attributable to its larger pyroelectric coefficient (*P*_c_ ≈ 436–524 µC/m^2^·K) compared to that of BTO (*P*_c_ ≈ 225–259 µC/m^2^·K). A similar trend is observed in PZT-based devices. Zhang et al. reported a typical PZT film-based PyNG [57]. When subjected to a temperature change from room temperature (23 °C) to a higher temperature (38 °C), the output *V*_oc_ and *I*_sc_ of the PZT-based PyNG reach approximately 100 V and 480 nA, respectively, under positive connection conditions. The high output of the PZT-based PyNG is governed by its large pyroelectric coefficient and the sizable electrode area, with both factors serving as linear multipliers of the current according to *I* = *P*_c_·*A*(*dT*/*dt*). Moving to polymer-based materials, Lee et al. reported a P(VDF-TrFE)-based PyNG [64]. Under a temperature change from room temperature to a higher temperature, the peak output *V*_oc_ and *I*_sc_ density of the P(VDF-TrFE)-based PyNG are approximately 2.5 V and 570 nA/cm^2^, respectively. A significantly higher output is achieved with PVDF-based devices. Figure 9c,d show the output *V*_oc_ and *I*_sc_ of a PVDF film-based PyNG [72]. When exposed to hot water at 80 °C, the peak output *V*_oc_ and *I*_sc_ of the PVDF-based PyNG reach 192.6 V and 12 μA, respectively. This remarkable performance stems predominantly from the high heating rate of hot water immersion.

Li et al. investigated the pyroelectric output of PVDF at different temperatures and the effect of the number of plasma layers on pyroelectric properties [73]. They reported that as PVDF is heated from 35 °C to 155 °C (at intervals of 10 °C), the current intensity gradually increases. This is consistent with the linear *I*–Δ*T* relationship in *I* = *P*_c_·*A*(*dT*/*dt*), where larger temperature excursions generate more polarization charge. They also found that the pyroelectric output of the device is enhanced with an increase in the number of plasma layers, reaching its peak at four layers, corresponding to a current signal of 18 nA. However, with five plasma layers, a drop in current intensity was observed, likely due to light loss caused by the finite penetration depth of the incident light. When no plasma layers are present, no temperature change occurs in PVDF because the thermal energy-harvesting device is absent, resulting in zero pyroelectric output. Gokana et al. developed a screen-printed SRE PyNG based on a 7 wt% Cs_0.33_WO_3_/PVDF composite [68]. They reported that the device exhibits an electrical output response of 4.36 V and 214 nA at 121 °C, with a power density of 23.28 μW/m^2^ at a load resistance of 20 MΩ. Compared to pure PVDF, which produced only 3.47 V and 185 nA, the Cs_0.33_WO_3_/PVDF fiber film achieved an increase of 26% in voltage and 16% in current. Furthermore, under the same near-infrared radiation, the temperature change rate of the 7 wt% Cs_0.33_WO_3_/PVDF pyroelectric device is 27% higher than that of pure PVDF, leading to a significantly higher electrical output. Niu et al. developed a pyroelectric device based on CIPS [34]. They observed that the rise and fall in temperature generate positive and negative current pulses, respectively, and that the rate of temperature change directly affects the peak output current. Critically, they revealed a strictly linear correlation between the electrode area and the output current. This observation provides a direct experimental validation of the pyroelectric equation *I* = *P*_c_·*A*(*dT*/*dt*). The linear *I*–*A* dependence (with a constant *P*_c_ and *dT*/*dt*) also offers a practical guideline for device design. Specifically, increasing the electrode area is an effective strategy to boost output, provided that the thermal uniformity across the enlarged area is maintained.

To systematically compare the output performance of various PyNGs, we summarize their key parameters in Table 2. Based on Table 2, the output performance of PyNGs varies considerably depending on the material system, device architecture, and excitation conditions. This disparity likely arises from differences in the pyroelectric coefficient, the electrode area, and the thermal coupling efficiency between the pyroelectric layer and the surrounding environment.

In summary, the output performance of PyNGs varies significantly depending on the material system and device architecture. Among ceramic-based materials, PZT film-based PyNGs exhibit the highest output voltage (approximately 100 V), while ZnO and KNbO_3_-based devices produce relatively lower voltages in the millivolt range, consistent with their much lower *P*_c_. In contrast, polymer-based materials, particularly PVDF, demonstrate superior output performance, achieving a peak voltage of 192.6 V and a current of 12 μA under optimal conditions, even though their intrinsic *P*_c_ is substantially lower than that of ceramics. This apparent contradiction is resolved by the equation *I* = *P*_c_·*A*(*dT*/*dt*), as the larger temperature variation applied to PVDF generates a sufficiently high *dT*/*dt* to compensate for its lower *P*_c_. Furthermore, performance enhancement strategies such as doping with functional materials and optimizing the number of plasma layers have proven effective in improving both voltage and current outputs. Notably, the CIPS-based device reveals a linear correlation between electrode area and output current, offering a design guideline for future device optimization. Overall, while ceramic materials provide high voltage output, polymer-based PyNGs offer greater flexibility and comparable or even higher performance, making them promising candidates for practical applications in wearable and self-powered electronic systems.

### 3.3. The Output Performance of the HNGs

The pyroelectric current equation can be extended to HNGs, though the situation becomes more complicated. In a hybrid device, we cannot simply add up the contributions from each mechanism—pyroelectric, piezoelectric, triboelectric, or photovoltaic—because they respond on different timescales and share the same electrodes. Even so, the pyroelectric part still follows the same equation.

**Figure 10:**
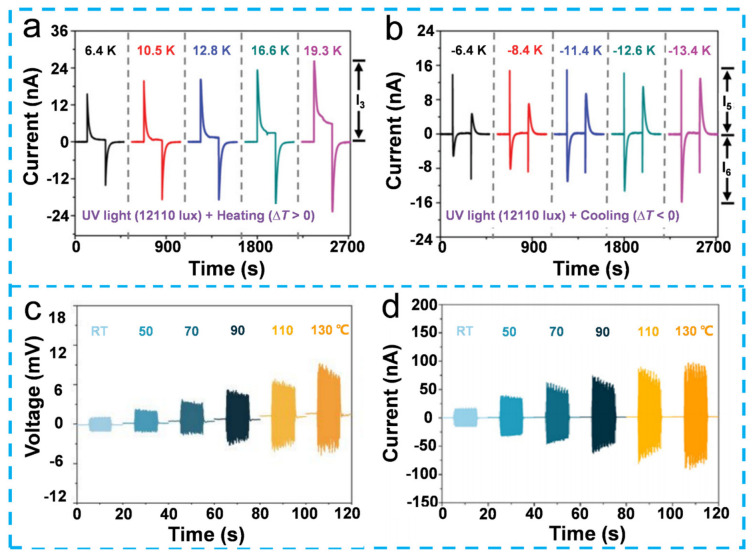
(**a**,**b**) The output currents of the BNT-BZT-based HNG under UV light irradiation and temperature changes. Reprinted with permission from Ref. [56]. (**c**,**d**) The output voltage and current of the BTO-based HNG under vibration excitation at different temperatures. Reprinted with permission from Ref. [58].

Zhao et al. leveraged the relationship between pyroelectric output and UV light intensity to design a temperature sensor with UV light regulation [56]. As shown in Figure 10a,b, compared with a pure pyroelectric system (without UV light coupling), the peak currents under the coupled operating conditions of “UV light + heating” and “UV light + cooling” exhibit significant differences. Specifically, the current peak under the “UV light + heating” mode is 88.6% higher, whereas that under the “UV light + cooling” mode is 37.3% lower than the corresponding values observed in the pure pyroelectric system. The designed temperature sensor was exposed to UV light at a wavelength of 395 nm. Under these conditions, it achieved detection sensitivities of 0.9 nA/K during the heating process and 1.48 nA/K during the cooling process, demonstrating asymmetric response characteristics. Figure 10c,d demonstrate the operation of the ITO-BTO-LNO HNG under varying temperature conditions, revealing a significant enhancement in its output performance as the temperature increases [58]. The device operates over a temperature range extending from room temperature to 130 °C, which exceeds the *T*_c_ of BTO (approximately 118 °C). This finding indicates that the nanogenerator can function effectively even above the phase transition point of the pyroelectric material. Furthermore, when the device is subjected to mechanical vibration excitation at elevated temperatures, the maximum voltage and current exhibit substantial increases. Specifically, the *V*_oc_ improves by 435%, and the *I*_sc_ improves by 400%, compared to the values measured under room temperature conditions.

Zhao et al. developed a multi-effect coupled nanogenerator capable of effectively responding to light, pressure, and temperature stimuli, with the ability to distinguish between them based on the waveform of the output current [74]. They reported that the device exhibits a detection sensitivity of 0.42 nA/(mW/cm^2^) under 405 nm light irradiation, a pressure detection sensitivity of 1.43 nA/kPa, and a temperature sensing sensitivity of 8.85 nA/K. Liu et al. investigated the performance of a photoelectric and pyroelectric coupler device under three different temperature conditions: room temperature (Δ*T* = 0 K), high temperature (Δ*T* = 18.3 K), and low temperature (Δ*T* = −17 K) [75]. They observed that under light irradiation, both the peak photocurrent (*I*_peak_) and the plateau photocurrent (*I*_platform_) increase with increasing temperature and decrease with decreasing temperature. This finding confirms the influence of temperature on the photoelectric properties. Specifically, at higher temperatures, the carrier mobility increases, the band gap of BNT becomes narrower, and electrons migrate more easily into the conduction band, leading to an increased carrier concentration. By changing the temperature, the device achieved a 131% enhancement in the peak photocurrent and a 57% enhancement in the plateau photocurrent when both light irradiation and heating were applied simultaneously, compared to the photocurrent generated by illumination alone.

**Figure 11:**
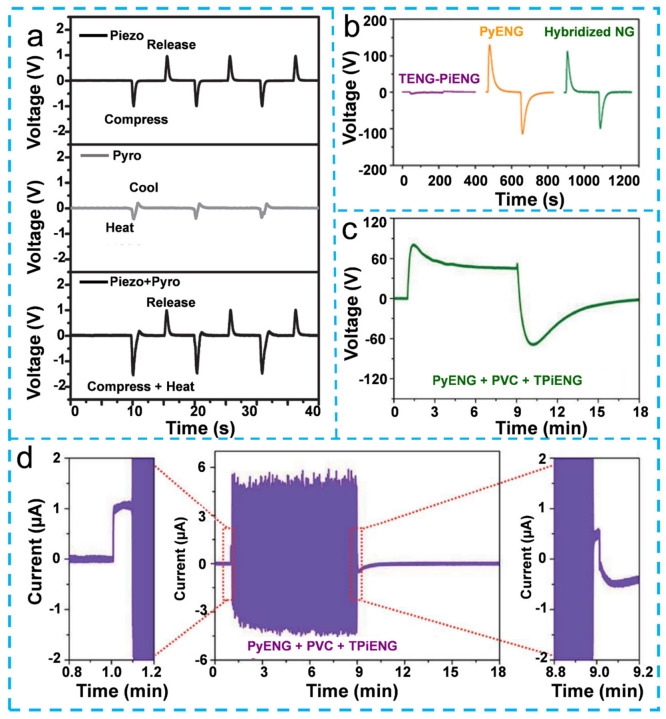
(**a**) The output voltages of P(VDF-TrFE)-based piezoelectric, pyroelectric and hybrid nanogenerators. Reprinted with permission from Ref. [65]. (**b**) The output voltages of PVDF film-based triboelectric–piezoelectric, pyroelectric and hybrid nanogenerators. Reprinted with permission from Ref. [37]. (**c**,**d**) The output voltage and current of the PZT-based HNG. Reprinted with permission from Ref. [57].

Figure 11a presents a comparison of the output voltages of P(VDF-TrFE)-based PENG, PyNG and HNG under different operating conditions [65]. Specifically, under compression-release conditions, the P(VDF-TrFE) PENG generates an output voltage of approximately 1.0 V. In contrast, the output voltage of the PyNG alone (without additional coupling) is about 0.4 V, while the HNG, which integrates multiple energy-harvesting mechanisms, achieves a significantly higher output voltage of approximately 1.4 V. This indicates that the hybrid design effectively enhances the overall electrical output. Figure 11b illustrates the output voltages of a PVDF-based PyNG and HNG [37]. The TENG-PiENG component produces a *V*_oc_ of approximately 5 V. Remarkably, the PyNG alone generates a much higher output voltage of about 120 V. However, when operating in the hybrid mode, the output voltage is slightly lower than 120 V. Specifically, frictional heating generated by the vibrating Nylon film in the TENG creates a thermal gradient opposite to the PyNG cooling process, which in turn generates a pyroelectric signal of reversed polarity. This negative signal, mediated through the shared electrode interface, induces charge redistribution that alters the local electric field distribution, while the triboelectric charges accumulated on the electrode surface simultaneously partially screen the pyroelectric polarization. As a consequence, the reversed-polarity signal partially cancels the dominant positive PyNG output, resulting in a reduced net voltage [37]. Figure 11c,d present the output voltage and current of a PZT film-based HNG, respectively [57]. The HNG, which integrates pyroelectric, piezoelectric, photovoltaic, and triboelectric effects (denoted as PyNG + PVC + TPiENG), achieves a peak output voltage of approximately 80 V and a peak output current of about 5 μA. These values demonstrate the capability of the PZT-based multi-effect coupling design to deliver substantial electrical output from combined energy sources.

The output performance of representative HNGs based on different material systems and multi-effect coupling strategies under various working conditions are summarized in Table 3. Notably, multi-effect coupling does not consistently enhance output, with some cases showing improvement while others exhibit a decline.

In conclusion, the output performance of HNGs is governed by the synergistic coupling of multiple physical effects, including pyroelectric, piezoelectric, triboelectric, and photovoltaic mechanisms. The integration of optical and thermal stimuli enables asymmetric response characteristics, providing new opportunities for designing advanced sensors with distinct heating and cooling detection modes. Multi-effect coupled nanogenerators have shown the capability to distinguish between different external stimuli based on the waveform of the output current, highlighting their potential for multi-functional sensing applications. Among polymer-based HNGs, the hybrid design generally outperforms single-effect devices, although careful engineering is required to mitigate potential trade-offs or coupling losses that may arise when integrating multiple energy-harvesting mechanisms. Notably, ceramic-based multi-effect HNGs achieve substantial electrical outputs by effectively combining pyroelectric, piezoelectric, photovoltaic, and triboelectric effects. Overall, HNGs offer significant advantages over single-effect devices through synergistic coupling, but optimal performance relies on rational device architecture design to minimize adverse interactions between different energy conversion mechanisms.

In assessing the energy-harvesting performance of pyroelectric devices, a note on energy conversion efficiency (η) is warranted. The Carnot efficiency sets the thermodynamic upper bound for any heat-to-electricity conversion process, while the Olsen cycle, comprising two isothermal and two isoelectric field processes, provides a more practical evaluation framework specifically for ferroelectric pyroelectric materials [7]. However, quantitative efficiency data remain remarkably scarce in the PyNG literature. The vast majority of reported studies focus on *V*_oc_ and *I*_sc_ without characterizing the thermal input power or calculating the conversion efficiency. A rare exception is the work by Kang et al. [76], who developed a dedicated testing setup to simultaneously measure heat flux and electrical output, and reported pyroelectric conversion efficiencies on the order of 3.19 × 10^−4^ for P(VDF-TrFE)-BTO composite PyNG, a value orders of magnitude below the Carnot limit.

The scarcity of efficiency reporting can be attributed to several factors: the difficulty in accurately measuring the thermal input to nanoscale or thin-film active layers, and the common practice of prioritizing electrical output characterization while neglecting the thermal measurements needed for conversion efficiency. We highlight this as a critical gap and recommend that future studies adopt the Olsen cycle framework for efficiency reporting to enable meaningful cross-device comparisons.

### 3.4. Applications of PyNGs and HNGs

Driven by the demand for distributed energy in fields such as the IoT, wearable electronics, and health monitoring, the practical applications of PyNGs and HNGs have expanded significantly.

Nevertheless, benchmarking the output capability of PyNGs against competing thermal harvesters is essential. Unlike TEGs, which harvest steady-state temperature gradients via the Seebeck effect, PyNGs rely on temporal temperature fluctuations. This distinction has profound implications for power delivery. In body heat harvesting, ambient temperature changes are typically slow, causing PyNGs to produce intermittent spikes rather than the continuous baseline power of TEGs. While wearable TEGs have demonstrated power densities orders of magnitude higher than those of PyNGs under steady-state gradients, most PyNGs deliver only nW-to-μW average outputs, which fall far short of the mW-level sustained power required by typical IoT sensor nodes. This performance gap does not render PyNGs irrelevant but redefines their role: rather than primary power sources, they are better suited as energy supplements that harvest otherwise wasted thermal fluctuations to extend battery life or enable duty-cycled operation of ultra-low-power sensors. In this role, they offer unique advantages, such as mechanical flexibility and form-factor adaptability that TEGs cannot readily match. Yet for continuous IoT power delivery, they remain complementary to batteries or TEGs, not replacements for them.

Beyond output performance, PyNGs also show promising potential for wearable health monitoring. In such applications, material safety becomes a critical consideration. The use of lead-based materials like PZT raises concerns that are difficult to ignore. While PZT-based PyNGs and HNGs offer impressive electrical outputs, their potential cytotoxicity and environmental risks should not be overlooked in practical use. With this in mind, the long-term development of wearable pyroelectric devices would benefit from a shift toward lead-free and biocompatible material systems.

**Figure 12:**
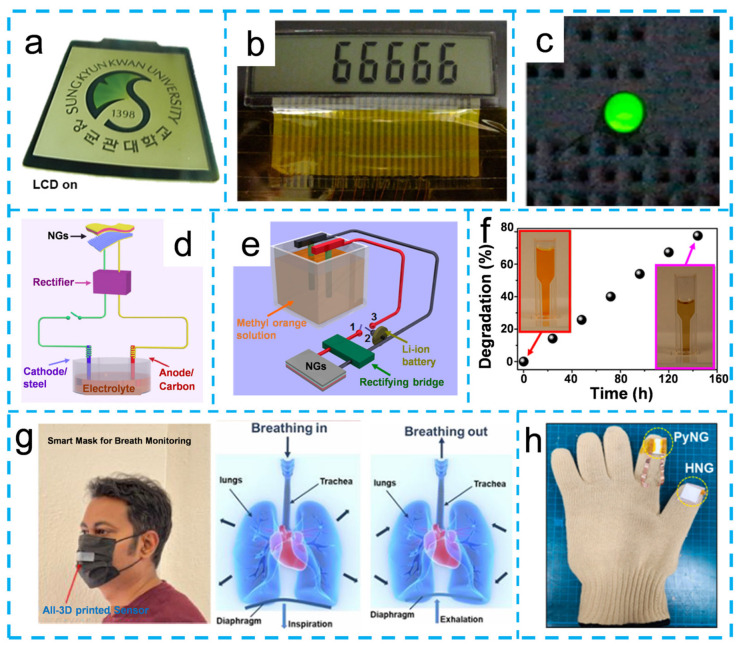
Applications of PyNGs and HNGs. (**a**,**b**) A series of LCDs powered by PyNG and HNG. Reprinted with permission from Refs. [64] and [25] respectively. (**c**) A green LED lighted by PyNG. Reprinted with permission from Ref. [33]. (**d**) Self-powered cathodic protection. Reprinted with permission from Ref. [61]. (**e**,**f**) Electrocatalytic deposition and methyl orange degradation. Reprinted with permission from Ref. [60]. (**g**) Smart mask-based breath monitoring. Reprinted with permission from Ref. [78]. (**h**) Intelligent glove for object recognition. Reprinted with permission from Ref. [80].

Figure 12a shows a photo of a black LCD with the Sungkyunkwan University logo lighting up after a stretchable PyNG charges the capacitor [64]. Upon hand touching, both a temperature rise and a compressive strain are generated in the PVDF film, and the instantaneous output from this dual-mode nanogenerator was used to power an LCD, as demonstrated in Figure 12b [25]. Although the output of PyNGs and HNGs generally in the μW range, falls short of sustaining continuous operation, it proves entirely sufficient, when coupled with a storage capacitor, to support intermittent operation. Gao et al. demonstrated that the electrical energy generated by a PyNG can be stored in a capacitor and subsequently used to power a smart electronic watch [52]. Similarly, Figure 12c demonstrates that a lithium-ion battery can be effectively charged using a PyNG, and the charged battery is subsequently capable of powering a green LED light [33]. The electrical energy generated by the tribo-piezo-pyroelectric HNG can be used for self-powered cathodic protection to prevent metal corrosion [61], as shown in Figure 12d. As shown in Figure 12e, integrating pyroelectricity with a TENG allows energy to be rectified and stored in a lithium-ion battery, forming a hybrid energy cell that degrades a methyl orange (MO) solution [60]. Figure 12f reveals the MO degradation progress; after 144 h, the degradation rate reaches 80%, evidenced by the solution becoming noticeably lighter. Zhao et al. developed a 3-by-3 sensor array using a BTO-based PyNG packaged in flexible PDMS [74]. By simultaneously applying 405 nm light and ice cooling to the second channel, the hybrid output performance was improved, showing promise for electronic skin and photodetectors. Li et al. demonstrated the application of PyNG in wearable devices, specifically integrating them into bracelets and health trackers [77]. They reported that a PyNG triggered by sunlight could charge a human health tracker to 10% of its maximum capacity. Furthermore, after one hour of outdoor exercise, the device was able to detect changes in the user’s heart rate. This is a promising step, but it also shows that PyNGs are about energy storage over time, not real-time power. In practice, they will need to be paired with energy storage and proper power management to make their intermittent output useful for practical loads. Figure 12g demonstrates the application of PyNG in human health monitoring, presenting a self-powered respiratory monitoring system based on a fully 3D-printed sensor-integrated mask [78]. Similarly, Xue et al. demonstrated that the electric energy generated by the PyNG, driven by the heat from human respiration, is rectified and stored in a capacitor. This stored energy can subsequently light up an LCD and power eight LEDs, demonstrating the application potential of PyNGs in wearable devices [79]. Figure 12h illustrates the HNG incorporating piezoelectric, triboelectric, and pyroelectric effects (Piezo-Tribo-Pyro) integrated into a glove. The device touches various objects and records the corresponding electrical outputs. Signals are collected from objects at different temperatures. The pyroelectric component aids in temperature detection, while the piezoelectric–triboelectric hybrid device measures output changes caused by triboelectric charging due to friction between the composite material and object surfaces [80].

In summary, the versatility of PyNGs and HNGs has led to their broad application across diverse fields. They are instrumental in powering optoelectronic devices (LCDs, LEDs), enabling self-powered systems (cathodic protection, environmental remediation), and advancing wearable technology (smartwatches, health trackers, respiratory monitors, and electronic skin). As research continues to improve their output performance and integration strategies, PyNGs and HNGs hold great promise for becoming a cornerstone technology in the rapidly growing sectors of the Internet of Things, personalized healthcare, and intelligent sensing.

## 4. Conclusions

This review systematically summarizes recent research advancements in PyNGs and HNGs. Optimizing the performance of pyroelectric materials involves enhancing both the densification of the material and the pyroelectric coefficient. The performance of PyNGs is critically influenced by the intrinsic pyroelectric coefficient of the material, along with device structure optimization and electrode material innovation. Beyond device-level optimization, we further distinguish the respective contributions of primary, secondary, and tertiary pyroelectric effects to the overall output performance. Furthermore, HNGs, which couple pyroelectric with piezoelectric, triboelectric, and photovoltaic effects can enhance output performance and harvest multiple energy sources simultaneously. In terms of material selection, ceramic-based materials offer superior pyroelectric coefficients, but their rigidity limits their use in wearable applications. In contrast, polymer-based materials provide flexibility and biocompatibility, albeit with lower pyroelectric coefficients.

Despite significant progress, several fundamental challenges still hinder the practical application of PyNGs, and addressing them will require coordinated interdisciplinary efforts. First, key processes such as polarization switching, domain wall motion, and charge trapping at interfaces during rapid thermal cycling remain largely invisible to conventional electrical measurements. Developing in situ or operando techniques to observe these dynamics is therefore essential for understanding the actual working mechanism of PyNGs. Second, for polymer-based devices, the effects of cyclic thermal loads and mechanical bending on polarization retention are largely unknown. Systematic fatigue tests under combined thermal and mechanical stress, paired with structural analysis, are needed to establish degradation mechanisms. Third, for PyNGs, harvesting ambient temperature fluctuations to extend battery life in low-power applications requires power management circuits, thermal coupling structures to enhance the temperature change rate, and integration with supercapacitors or batteries for steady supply. Addressing these challenges will require collaboration across materials science, electrical engineering, and system design to turn PyNGs into a viable technology for battery-free wearable and IoT devices.

## Figures and Tables

**Table 1:** Summary of the pyroelectric coefficient, Curie temperature, and dielectric constant of various pyroelectric materials.

Material	Pyroelectric Coefficient, *P*_c_ (µC/m^2^·K)	Curie Temperature, *T*_c_ (°C)	Dielectric Constant, *ε*_r_	Ref.
ZnO	12–15	—	12.07	[27,43]
BTO	225–259	120	2000	[44,45]
BNT	436–524	320	1300	[46,47]
KNbO_3_	50	410	540	[29,48]
BFO	71	830	200	[49]
PMN-PT	1040	130	5000	[31,50]
PZT	800	320	600	[33,51]
PVDF	27.2	190	8	[52,53]
P(VDF-TrFE)	39	108	36	[36,39]

**Table 2:** Summary of PyNGs output performance.

Material	Device Architecture	Temperature Excitation Conditions (Δ*T* or *dT*/*dt*)	Output Performance	Power Density	Electrode Area	Ref.
ZnO NW	Ag/ZnO/ITO	9 K	5.8 mV, 120.4 pA	—	15 mm^2^	[27]
KNbO_3_ NW-PDMS	Ag/KNbO_3_-PDMS/ITO	40 K	10 mV, 120 pA	—	—	[29]
PMN-PT	Au/PMN-PT/Cr-Au	8 K	0.1 V, 20 nA	2 mW/cm^3^	9 mm^2^	[31]
BTO	ITO/BTO/Ag	0.98 K/s	2.9 V, 49.8 nA	3.5 nW/cm^2^	2.22 cm^2^	[44]
BNT	ITO/BNT/Ag	29.8 K	31.5 V, 0.261 μA	2.65 μW/cm^2^	2.01 cm^2^	[47]
PZT	AgNWs/PDMS-ITO/PZT/Ag	15 K	100 V, 480 nA	0.27 μW/cm^2^	49 cm^2^	[57]
P(VDF-TrFE)	Ag/AgNWs/P(VDF-TrFE)/Au	22 K	2.5 V, 570 nA/cm^2^	—	—	[64]
PVDF	Cu/PVDF/Cu	80 K	192.6 V, 12 μA	14 μW/cm^2^	9 cm^2^	[72]
PVDF	Au/PVDF/Graphene@AgNWs	130 K	9.1 V, 18 nA	—	—	[73]
Cs_0.33_WO_3_/PVDF	Graphene@AgNWs/Cs_0.33_WO_3_-PVDF/Ni-Cu	96 K	4.36 V, 214 nA	23.28 μW/m^2^	25 cm^2^	[68]
CIPS	Au/CIPS/Si	20 K	—, 350 pA	—	—	[34]

**Table 3:** Summary of HNG output performance.

Material	Device Architecture	Coupling Effects	Working Condition	Output Performance (Single-Effect)	Output Performance (Multi-Effect)	Electrode Area	Ref.
BNT-BZT	ITO/BNT-BZT/Ag	pyro-photoelectric	Δ*T* = 6.4 K/−6.4 K, 12,110 lux	Heating: 8.2 nA; Cooling: −8.3 nA	Heating: *I*_sc_ ↑ 88.6%; Cooling: *I*_sc_ ↓ 37.3%	69.2 mm^2^	[56]
BTO	ITO/BTO/LNO/Mica	pyro-mechanical vibration	Δ*T* = 105 K, 15 Hz	1.2 mV, 17 nA	*V*_oc_ ↑ 435%, *I*_sc_ ↑ 400%	24 mm^2^	[58]
BTO	ITO/BTO/Ag	pyro-photoelectric	Δ*T* = −19.5 K, 83.2 mW/cm^2^	21.6 nA	*I*_sc_ ↑ 375%	78.5 mm^2^	[74]
BNT	ITO/BNT/Ag	pyro-photoelectric	Δ*T* = 18.3 K, 156.05 mW/cm^2^	*I*_peak_: 0.86 µA, *I*_platform_: 0.60 µA	*I*_peak_ ↑ 131%, *I*_platform_ ↑ 57%	2.54 cm^2^	[75]
P(VDF-TrFE)	Graphene/P(VDF-TrFE)/PDMS-CNT	pyro-piezoelectric	compress-release + Δ*T*	*V*_PENG_: ~1.0 V, *V*_PyNG_: ~0.4 V	~1.4 V	—	[65]
PVDF	PDMS-PVDF/ITO/PVDF/ITO	tribo-piezo-pyroelectric	Δ*T* = −11 K, air flow at 15 m/s	*V*_PyNG_: 120 V	*V*_oc_: slight decrease	—	[37]
PZT	Nylon/FEP/AgNWs/PDMS-ITO/PZT/Ag	pyro-photo-triboelectric	Δ*T* = 15 K, 15 m/s, light	*I*_PyNG_: 480 nA, *I*_PVC_: 890 nA, *I*_TPiENG_: 3.8 µA	*I*_sc_: ~5 µA	49 cm^2^	[57]

## Data Availability

No new data were created or analyzed in this study. Data sharing is not applicable to this article.
